# Migration of Plastic Additives and Non-Intentionally Added Substances from Packaging into Edible Oils and Beverages: A Combined GC–MS and Hydrolysis–Capillary Electrophoresis Approach

**DOI:** 10.3390/polym18080965

**Published:** 2026-04-15

**Authors:** Rodica Sturza, Veronica Dragancea, Aliona Ghendov-Mosanu, Ileana-Denisa Nistor, Diana-Carmen Mirila, Dmitri Lazacovici

**Affiliations:** 1 Department of Oenology and Chemistry, Faculty of Food Technology, Technical University of Moldova, 9/9 Studentilor Street, MD-2045 Chisinau, Moldova; rodica.sturza@chim.utm.md (R.S.); dlazakowich@outlook.com (D.L.); 2 Department of Food Technology, Faculty of Food Technology, Technical University of Moldova, 9/9 Studentilor Street, MD-2045 Chisinau, Moldova; aliona.mosanu@tpa.utm.md; 3 Department of Chemical and Food Engineering, Faculty of Engineering, “Vasile Alecsandri” University of Bacau, 157 Calea Marasesti, 600115 Bacau, Romania; dnistor@ub.ro (I.-D.N.); miriladiana@ub.ro (D.-C.M.)

**Keywords:** phthalate esters, aqueous-alcoholic matrices, capillary electrophoresis, *o*-phthalic acid, hydrolysis method, beverage contamination

## Abstract

The present study aimed to investigate the migration of potentially hazardous compounds from plastic food packaging into edible oils, bottled water and soft drinks available on the market in the Republic of Moldova. GC–MS screening was applied to identify plastic additives and unintentionally added substances (NIAS). The influence of key extraction parameters, including solvent type, extraction time, pH, alcohol content and sugar concentration, was systematically investigated. The optimized procedure demonstrated satisfactory analytical performances, with recoveries ranging from 81 to 96%, repeatability below 5% and detection limits between 0.006 and 0.01 mg/L. To allow a comprehensive assessment of total phthalate contamination, an additional analytical approach based on the hydrolysis of phthalate esters and the determination of *o*-phthalic acid using capillary electrophoresis with spectrophotometric detection was proposed. The method showed a linearity range of 0.1–5.0 mg/L and a limit of quantification of 0.07 mg/L. The combined chromatographic and hydrolysis-capillary electrophoresis approaches provide a reliable tool for the integrated determination and evaluation of phthalate residues in aqueous-alcoholic systems and beverages, accessible to laboratories performing food quality control.

## 1. Introduction

Over recent decades, human dietary patterns have undergone significant changes, shifting from the consumption of seasonal and locally sourced foods to an increasing reliance on products transported and stored over long distances, driven by urbanization and industrialization [1]. Concurrently, the demand for pre-packaged food products has increased substantially [2]. As a result, the food packaging industry has evolved into a dynamic and indispensable sector of the global economy.

Plastics dominate the packaging industry due to their low cost, lightweight nature, flexibility, and favorable barrier properties, accounting for approximately 52% of local packaging markets [3]. The most widely used polymers include polypropylene (PP), polyethylene (PE), polyvinyl chloride (PVC), polystyrene (PS), polyethylene terephthalate (PET), and polyurethane. These materials are processed into various forms, such as films, trays, and bottles, through the application of heat and pressure, often in combination with specific additives that impart the required mechanical and functional properties [4,5,6].

However, plastic food packaging exhibits significant limitations in its ability to protect food, particularly in the absence of additives [7,8]. In addition, chemical migration represents a major concern: substances originating from the polymer matrix, including residual monomers, may migrate into food throughout the entire life cycle of the packaging material, especially under the influence of physicochemical factors such as temperature, oxygen exposure, and humidity [9,10]. Monomers and plasticizing additives, such as Bisphenol A, Styrene, and Phthalates, have been associated with adverse health effects, including endocrine system disruption. These effects are of particular concern for vulnerable populations, such as pregnant women and children [11,12,13].

It is estimated that approximately 10,000 chemical substances may migrate from plastic materials into food, yet only a small fraction of these compounds has been thoroughly studied. To date, over 2000 substances lack complete toxicological profiles, and 1327 chemicals remain insufficiently regulated at the global level [14,15,16]. This underscores the need for extensive research to assess potential risks to human health and to ensure that food packaging materials effectively maintain their primary function of protecting food products.

The migration of chemical substances from packaging materials into food represents a complex phenomenon, which can be categorized into two main types: overall migration and specific migration [17]. Overall migration quantifies the total amount of substances transferred from the packaging into food without distinguishing individual components, whereas specific migration refers to particular, identifiable compounds—such as plasticizers or additives—for which regulatory limits are established for each individual component [18,19]. The assessment of migration involves exposing packaging materials to standardized conditions of time and temperature to evaluate the amount of compounds transferred. Due to the challenges associated with testing using real foods, these measurements are typically conducted with food simulants that mimic the properties of different food types—such as fatty, acidic, liquid, or solid matrices—enabling standardized evaluations [20,21].

The migration of substances is governed by processes such as diffusion, partitioning, and material permeability, affecting compounds including plasticizers, antioxidants, and stabilizers, which are added to enhance the durability of packaging materials. Factors influencing packaging–food interactions include the chemical properties of the polymers, food composition, temperature, and contact duration. Fatty or acidic foods promote the migration of certain compounds, such as Bisphenol A and Phthalates, which are known for their adverse health effects [11,22].

Plastic materials invariably contain residual monomers at low concentrations (0–2%) due to incomplete polymerization [23].

Low-molecular-weight substances tend to form branched structures, facilitating the diffusion of monomers throughout the polymer matrix. Although polymer-bound monomers are generally stable and non-toxic, interactions with food can convert them into harmful compounds, leading to increased concentrations in the human body [24,25]. Phthalic acid esters (PAEs), with a global annual production of several million tons, have become ubiquitous contaminants in both the environment and the food chain [26]. Biomonitoring studies in the United States (NHANES) indicate that urinary metabolites of dibutyl phthalate (DBP) and di(2 ethylhexyl) phthalate (DEHP) are detected in nearly all participants, reflecting widespread exposure [27]. Phthalate exposure has been linked to endocrine disruption and multiple adverse health effects, including reproductive, developmental, hepatic, and cardiovascular toxicity in both experimental and epidemiological studies [28,29,30]. Due to their lipophilic nature, phthalic acid esters (PAEs) tend to accumulate in lipid-rich food matrices, particularly in edible oils and other high-fat products [31,32].

Regulatory frameworks have responded to concerns about phthalate migration. In the European Union, Regulation (EU) 10/2011 sets specific migration limits for DBP (≤0.12 mg/kg food) and DEHP (≤0.60 mg/kg food), and a combined migration limit for several phthalates [33]. Certain phthalates, including DBP and DEHP, are also listed as priority pollutants by the United States Environmental Protection Agency due to their environmental persistence and toxicity [34].

PET, widely used for beverage bottling due to its relative inertness and absence of plasticizers, has nonetheless been shown to release a wide range of substances under real conditions. Systematic analyses revealed that, among 193 chemicals investigated in PET bottled beverages, 150 substances were detected including phthalates and metal residues, with 18 exceeding EU regulatory limits in specific food matrices [35,36].

Many of these substances are classified as non-intentionally added substances (NIAS), which include impurities, reaction by products, degradation products, and contaminants introduced during processing or recycling. The complexity of polymer formulations, processing conditions, storage environments, and beverage compositions contributes to the incomplete characterization of NIAS [18,37,38]. From an analytical perspective, identification and quantification of NIAS are challenged by the lack of analytical standards and comprehensive mass spectral databases, often necessitating semi-quantitative approaches [39,40,41].

Several recent studies have employed advanced analytical technologies for the investigation of migrants and NIAS from polymeric packaging. For example, gas chromatography–mass spectrometry (GC–MS) non-targeted screening has been used to identify potential chemical migrants in candy wrappers and other packaging matrices [42]. Thermal desorption GC–MS has been applied for evaluating phthalate migration in polymer systems [43]. In addition, migration of a broad spectrum of chemical migrants from PET food packaging has been reported in market samples [44].

Understanding the migration of chemical additives from polymeric packaging materials is essential not only for food-safety assessment but also for the development of safer and more sustainable packaging systems. Despite increasing attention to additive migration, significant analytical challenges remain in the comprehensive assessment of phthalates and other NIAS in complex food matrices. Conventional analytical approaches often target known compounds, potentially underestimating total phthalate contamination due to the presence of degradation products or metabolites.

In this context, the present study aimed to investigate the migration of potentially hazardous compounds from plastic food packaging into edible oils, bottled water, and soft drinks available on the market in the Republic of Moldova. GC–MS screening was applied to identify plastic additives and NIAS, including phthalates, styrene oligomers, fatty amides, and flame retardants. In addition, an analytical procedure for phthalate determination was optimized and validated. Furthermore, a complementary approach based on alkaline hydrolysis of phthalate esters followed by determination of *o*-phthalic acid using capillary electrophoresis was developed to enable a more comprehensive estimation of total phthalate contamination in food matrices.

## 2. Materials and Methods

### 2.1. Sample Collection and Characterization

A total of 65 commercially available products packaged in plastic containers were collected from retail outlets and commercial suppliers, including still water (n = 14), carbonated water (n = 14), soft drinks (n = 26), and vegetable oils (n = 11). The polymer type of each container was identified using Resin Identification Codes. Packaging labels were examined for the presence of recycling symbols, food contact symbols (fork and cup pictogram), the Tidyman symbol, and any instructions on storage conditions or single-use status.

For migration experiments, selected samples included three still water containers, two carbonated water containers, two soft drink containers, and six vegetable oil containers, all obtained from different manufacturers. All samples had been bottled at least 60 days prior to analysis to ensure representative migration behavior.

### 2.2. Reagents

Analytical standards were purchased from Fluka and Sigma–Aldrich (PESTANAL^®^, Sigma–Aldrich, St. Louis, MO, USA) and included dimethyl phthalate (DMP, ≥99.6%), diethyl phthalate (DEP, ≥99.0%), dibutyl phthalate (DBP, ≥99.8%), bis(2-ethylhexyl) phthalate (DEHP, ≥99.7%), dioctyl phthalate (DOP, ≥99.7%), and didecyl phthalate (DDP, ≥99.8%). A certified phthalate mixture (≥95.0%) and phthalic anhydride were also used. Aldrin solution (5000 μg/mL in methanol, Supelco, Bellefonte, PA, USA) was used as an internal standard for GC–MS analysis. Extraction solvents included chloroform (HPLC grade, 99.8%, Chem-Lab n.v., Zedelgem, Belgium), diethyl ether (analytical grade, Kuzbassorghim, Moscow, Russia), hexane (analytical grade, Reakhim, Moscow, Russia), carbon tetrachloride (≥99.8%, Ecos-1, Russia), benzene (analytical grade, Reakhim, Moscow, Russia), and isoamyl alcohol (analytical grade, Reakhim, Moscow, Russia).

Additional reagents included glacial acetic acid (HPLC grade, Millipore Sigma Supelco, Bellefonte, PA, USA), sodium sulfate (≥99.9%, Brenntag Polska, Kędzierzyn-Koźle, Poland), sodium chloride (analytical grade, Reakhim, Moscow, Russia), and sodium hydroxide (≥98.5%, Brenntag Polska, Kędzierzyn-Koźle, Poland), which was used for the preparation of model solutions and for the saponification of *o*-phthalic acid esters.

Hexane (HPLC grade, 95%, Chem-Lab n.v., Zedelgem, Belgium) and isopropanol (HPLC grade, 99.8%, Chem-Lab n.v., Zedelgem, Belgium) were used for rinsing glassware and laboratory equipment. Deionized water (conductivity < 0.05 μS/cm) was obtained using a Milli-Q Simplicity UV purification system (Millipore, Burlington, MA, USA).

### 2.3. Analytical Methods and Techniques

Solutions used for the analysis of component migration from plastic packaging were prepared in accordance with Commission Regulation (EU) 2016/1416, which amends Regulation (EU) No. 10/2011 on plastic materials and articles intended to come into contact with food (Off. J. Eur. Union 2016, L230, 22–40).

Migration test media included 10% (*v*/*v*) ethanol for still water, 3% (*w*/*v*) acetic acid for soft drinks and carbonated beverages, and HPLC-grade isopropanol (Chem-Lab, Zedelgem, Belgium) for vegetable oils.

Migration tests for non-intentionally added substances (NIASs) were performed using containers previously rinsed with HPLC-grade hexane and deionized water. The tests were conducted at room temperature (20–22 °C) for 30 days for aqueous matrices and soft drinks, and for 14 days in the case of oil-based media.

Extraction of analytes from liquid samples was carried out using a laboratory orbital shaker (PSU-20i, Biosan, Rīga, Latvia). The extractant-to-sample ratio was maintained at 10 mL per 100 mL of sample. Samples and extractants were mixed in 500 mL separatory funnels and shaken for 20 min at 170 rpm with an oscillation amplitude of 20 mm. Phase separation was achieved within approximately 20 min after agitation.

When stable emulsions were formed, phase coalescence was facilitated by ultrasonic treatment followed by filtration through filter paper containing approximately 10 g of anhydrous sodium sulfate to remove residual moisture.

The resulting filtrate was transferred into a 20 mL vial and spiked with aldrin as an internal standard. After thorough mixing, an aliquot of 1.5 mL of the extract was transferred to a GC vial for subsequent GC–MS analysis

### 2.4. Preparation of Calibration Solutions and Saponification

Stock calibration solutions of phthalates and *o*-phthalic acid were prepared in ethanol (96.3–96.5% *v*/*v*). Working calibration solutions were obtained by appropriate dilution of the stock solutions with the same solvent.

Saponification of *o*-phthalic acid esters was performed using a 0.125 M NaOH solution (0.5 g per 100 mL). After addition of the sample, the pH of the test solution was approximately 3.0, while the calculated pH of the NaOH solution was 13.1. In the resulting reaction mixture (100 mL of sample and 1.0 g NaOH), the final pH reached approximately 12.8. The total phthalate content in 100 mL of the test solution was estimated at 2.3 μmol.

Hydrolysis was carried out by heating the reaction mixture to 90–95 °C (below the boiling point) for 30 min. This heating time was experimentally determined as the minimum required to achieve complete saponification. After hydrolysis, the solution was cooled to room temperature and subsequently neutralized to pH 7.0 using 50% acetic acid.

### 2.5. GC–MS Analysis

Separation of analytes was performed using a fused silica capillary column Rtx-5MS (5% diphenyl–95% dimethylpolysiloxane, 30 m × 0.25 mm i.d., 0.25 μm film thickness, Restek, Bellefonte, PA, USA) installed in a GCMS-QP2010S system (Shimadzu, Kyoto, Japan).

Gas chromatography conditions were as follows: injector temperature 280 °C; oven temperature program: 50 °C (2 min), increased at 15 °C/min to 100 °C, then at 20 °C/min to 200 °C, and finally at 10 °C/min to 300 °C. Helium (99.999%) was used as the carrier gas at a constant column flow rate of 1.6 mL/min. Samples were injected in split mode (1:10). The interface temperature was maintained at 280 °C.

Mass spectrometry conditions included electron impact ionization (EI, 70 eV) in positive ion mode, with an ion source temperature of 250 °C and an interface temperature of 280 °C. Mass spectra were recorded in the scan range of *m*/*z* 19–600.

Phthalates were identified by comparing retention times and mass spectra with reference spectra from the NIST08 and FFNSC 1.2 libraries. Quantitative determination was based on characteristic ions obtained from the mass spectra: DMP (*m*/*z* 163, 164, 194), DEP (*m*/*z* 149, 177), DBP (*m*/*z* 149), DEHP (*m*/*z* 149, 167, 279), DOP (*m*/*z* 149, 279), and DDP (*m*/*z* 149, 307). Quantification was performed using peak areas and calibration curves constructed from standard solutions.

### 2.6. Analysis of O-Phthalic Acid by Capillary Electrophoresis

After completion of the hydrolysis process, excess alkali was neutralized with 50% acetic acid to obtain a neutral pH of approximately 7.0. The resulting solution was filtered through a 0.45 μm membrane filter or centrifuged at 4000 rpm for 5 min to remove suspended particles.

Capillary electrophoresis analysis was performed using a KAPEL-105M system. Separation was carried out in a buffer solution containing 10 mM benzoic acid, 9 mM diethylamine, 0.5 mM cetyltrimethylammonium bromide, and 0.1 mM disodium ethylenediaminetetraacetate. The electrodes were operated under negative polarity.

Selective detection of *o*-phthalic acid was performed using a spectrophotometric detector set at a wavelength of 200 nm, corresponding to the absorption maximum of the compound in the UV–Vis spectrum [45].

### 2.7. Statistical Analysis

All measurements were performed in triplicate. Differences between mean values were considered significant at *p* < 0.05. Statistical analysis was performed using Statgraphics Centurion XVI 16.1.17 (Statgraphics Technologies Inc., The Plains, VA, USA).

## 3. Results and Discussion

### 3.1. Identification of NIAS Migrating from Packaging

The present study investigated the migration of potentially hazardous compounds from plastic packaging materials used for bottled water, soft drinks, and edible vegetable oils commercially available on the market of the Republic of Moldova. Migration experiments were conducted using model solutions simulating contact with food matrices under controlled laboratory conditions.

**Figure 1:**
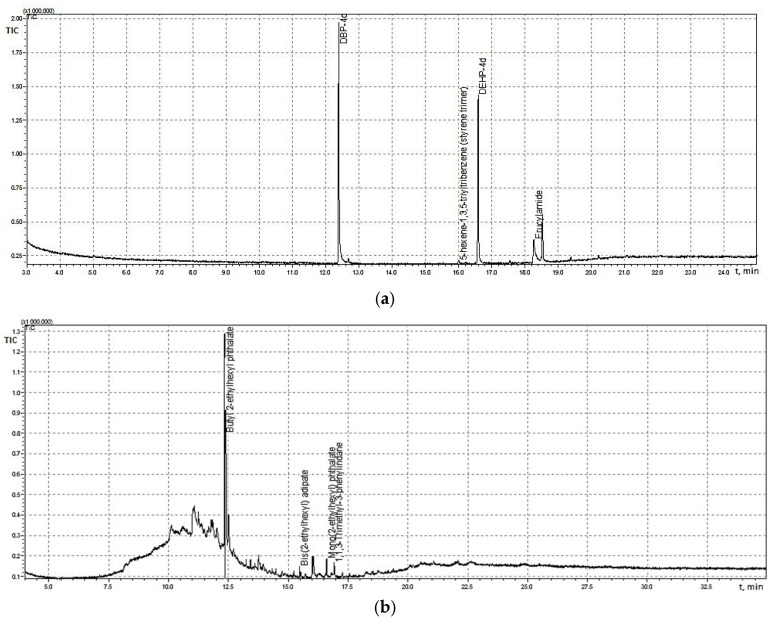
NIAS migration chromatograms from: (**a**) still water packaging, migration time—30 days, room temperature (20–22 °C), model solution—ethanol 10% (*v*/*v*); (**b**) sunflower oil packaging, migration time—14 days, room temperature; model solution—isopropanol, static regime.

Chromatographic analysis revealed the presence of several non-intentionally added substances (NIAS) originating from plastic packaging materials. In particular, relatively high levels of organophosphorus compounds were detected in some vegetable oil packaging samples. Among these compounds, phosphoric acid tris(2-ethylhexyl) ester (Disflamoll TOF) and 1,1,3-trimethyl-3-phenylindane (>80%) were identified in sunflower oil packaging samples (Figure 1).

Numerous studies have demonstrated that flame retardants can migrate from polymeric materials into food simulants and food products, particularly those with high lipid content [46]. Organophosphorus esters (OPEs) are widely used as plasticizers and flame-retardant additives in polymer manufacturing, including food packaging materials. Their widespread use has raised environmental and toxicological concerns due to their persistence and potential human exposure through dietary intake [47].

Food packaging materials have been recognized as an important source of OPE contamination in food products. However, the migration behavior of OPEs from plastic packaging into food matrices remains relatively poorly characterized. Previous work simulating migration from polypropylene food packaging into whole milk powder demonstrated that migration rates depend strongly on compound physicochemical properties, temperature, fat content of the receiving phase, and diffusion processes within the polymer matrix [48].

The migration potential of OPEs is strongly influenced by molecular weight and temperature. Low-molecular-weight OPEs (<300 Da) migrate significantly faster than higher-molecular-weight analogues. Migration efficiencies show a statistically significant negative correlation with molecular weight (*p* < 0.01) and a positive correlation with temperature (*p* < 0.01). Furthermore, higher migration levels are typically observed in fatty foods due to their increased affinity for lipophilic compounds [49].

This migration phenomenon is primarily attributed to the fact that such additives are not covalently bound to the polymer matrix. Instead, they are physically incorporated into the material and can therefore diffuse into contacting media during storage and use conditions. As a result, the consumption of contaminated food may represent a significant pathway of human exposure to these substances [50].

Due to concerns regarding toxicity and environmental persistence, brominated flame retardants are increasingly being replaced by organophosphate alternatives. However, the toxicological profiles of many OPEs remain insufficiently characterized. Their structural similarity to organophosphate insecticides has raised concerns regarding potential neurotoxicity and developmental toxicity. Experimental studies have shown that most investigated OPEs display measurable biological activity in vitro at concentrations between 1 and 10 μM, comparable to that of brominated flame retardants [51]. Epidemiological and animal studies have further associated certain OPEs with adverse health outcomes including endocrine disruption, neurotoxicity, carcinogenicity, and contact irritation [52].

Another compound detected in the analyzed samples was 1,1,3-trimethyl-3-phenylindane, which is classified as an irritant substance and has previously been identified in baking paper and paperboard materials intended for food contact applications [53].

Migration studies have reported concentrations of approximately 1.04 ± 0.11 µg/kg after migration from polystyrene trays into 95% ethanol, a commonly used simulant for fatty foods [54].

The detection of these compounds in vegetable oil packaging materials highlights the potential contribution of polymer packaging to chemical contamination of lipid-rich food products.

### 3.2. Migration of Phthalates from PET Packaging

The analysis of PET packaging used for bottled water and soft drinks revealed the presence of several phthalate plasticizers (Table 1). Among the quantified compounds, DBP and DEHP were detected in most analyzed samples.

These values are substantially lower than those reported for sunflower oil packaged in PET containers in Turkey, where phthalate concentrations ranged between 2.227 and 6.673 mg/dm^3^ [55]. Comparable studies on PET-bottled beer reported DBP and DEHP concentrations between 0.004–0.027 mg/dm^3^ and 0.005–0.058 mg/dm^3^, respectively [56].

In PET-bottled water, only DBP and DEHP were detected, with detection frequencies of 50% and 58% and average concentrations of 0.104 μg/dm^3^ and 0.082 μg/dm^3^, respectively [57].

Phthalate esters are among the most widely used plasticizers in polymeric materials, with global production reaching several million tons annually. The most commonly used compounds include DEHP, DBP, di-isononyl phthalate, and di-isodecyl phthalate [58].

Because phthalates are not chemically bound to polymer matrices, they can migrate into food products through diffusion processes, particularly in contact with fatty foods or oils. Migration levels may also increase during prolonged storage or repeated use of plastic containers. For instance, studies have reported increased DEHP migration from repeatedly refilled PET bottles, emphasizing the importance of regulatory restrictions on bottle reuse [59,60].

The European Food Safety Authority (EFSA) has established tolerable daily intake (TDI) values for several phthalates. Current EU regulations also define specific migration limits for food contact materials, including 1.5 mg/kg for DEHP, 0.3 mg/kg for DBP, 30 mg/kg for BBP, and 18 mg/kg for DEHA [56]. The concentrations measured in the present study were significantly below these regulatory thresholds.

Nevertheless, concerns remain regarding cumulative exposure to phthalates and their potential endocrine-disrupting effects. Mono-(2-ethylhexyl) phthalate, the primary metabolite of DEHP, has been associated with developmental toxicity and endocrine disruption. Epidemiological studies have shown correlations between prenatal exposure to phthalates and alterations in male reproductive development, including reduced anogenital distance and impaired androgen-dependent genital formation [61,62].

The detection of phthalates and related metabolites in vegetable oil packaging samples therefore highlights the need for continuous monitoring of endocrine-disrupting compounds in plastic food contact materials.

### 3.3. Migration of Polymer Additives and Oligomers

Trace amounts of 5-hexene-1,3,5-triyltribenzene (styrene trimer) and erucylamide were detected in five out of seven analyzed PET packaging samples used for bottled water and soft drinks (Table 1).

The migration behavior of PET oligomers and additives remains insufficiently studied, as many investigations rely primarily on model systems rather than real food matrices [63]. Long-term storage studies have shown that oligomer migration into edible oils stored in PET bottles may be limited under typical storage conditions. However, significant quantities of cyclic and linear PET oligomers have been detected in food simulants such as ethanol solutions as well as in both virgin and recycled PET containers [64].

Migration processes in PET are largely controlled by the polymer’s extremely low diffusivity. As a result, partition coefficients between polymer and food have only a limited influence on overall migration levels [65]. Higher-molecular-weight oligomers generally diffuse more slowly within the polymer matrix, although they may exhibit lower activation energies for diffusion processes [66].

Erucamide (CAS 112-84-5) is a primary fatty amide widely used as a slip agent during plastic processing. It is formed through the condensation of erucic acid with ammonia and is commonly applied in polyolefin packaging materials to reduce friction during manufacturing. Erucamide can migrate to the polymer surface and subsequently diffuse into contacting media, depending on storage conditions and surface distribution characteristics [67].

Alkylamides such as erucamide are widely used additives in plastic manufacturing and may represent emerging contaminants in food packaging systems. Comprehensive analytical investigations have identified more than 36 alkylamides in various polymeric materials, several of which were detected across multiple plastic types [68]. These findings highlight the importance of monitoring additive migration and potential environmental contamination pathways.

### 3.4. Implications for Dietary Exposure and Microplastic-Associated Contamination

Although definitive conclusions regarding the health risks of microplastic ingestion remain limited, public awareness of this issue has increased significantly in recent years [69]. Many NIAS compounds are lipophilic and may adsorb onto microplastic particles, thereby facilitating their ingestion through food and beverages.

Analyses of drinking water samples have revealed the presence of microplastics in both tap and bottled water. Reported concentrations ranged from up to 628 particles/L in tap water and 4889 particles/L in bottled water in European samples [70]. Based on average consumption patterns, annual adult exposure may reach approximately 458,000 microplastic particles from tap water and 3,569,000 particles from bottled water.

These findings highlight a potential link between polymer packaging materials, NIAS migration, and human dietary exposure. The combined presence of plastic additives, oligomers, and microplastic particles in food and beverages underscores the need for continued monitoring and improved risk assessment strategies for polymer-based food packaging materials.

### 3.5. Development and Validation of the Analytical Method

Reliable determination of phthalates in beverages and aqueous-alcoholic matrices requires analytical procedures capable of providing sufficient sensitivity, selectivity, and reproducibility. In the present study, a GC–MS method combined with liquid—liquid extraction was developed and optimized for the determination of phthalate esters in water, beverages, and alcoholic products. Particular emphasis was placed on achieving a cost-effective and robust analytical procedure suitable for routine laboratory monitoring.

The selection of an appropriate extraction solvent is essential for the efficient recovery of phthalate esters from aqueous beverage matrices. In this study, several organic solvents with different polarities were evaluated, including chloroform, carbon tetrachloride, benzene, diethyl ether, hexane, and isoamyl alcohol.

The extraction efficiency was investigated using a model solution containing six representative phthalates: DMP, DEP, DBP, DEHP, DOP, and DDP. Quantitative determination was performed by GC–MS using characteristic mass-to-charge ratios obtained from scanned mass spectra. The results presented in Table 2 indicate significant differences in extraction efficiency depending on the solvent used.

Diethyl ether and benzene showed the lowest extraction efficiency, whereas chloroform and carbon tetrachloride provided the highest recoveries for most phthalates. The improved performance of these solvents may be related to their relatively high density, which facilitates efficient phase separation and enhances analyte transfer into the organic phase.

**Figure 2:**
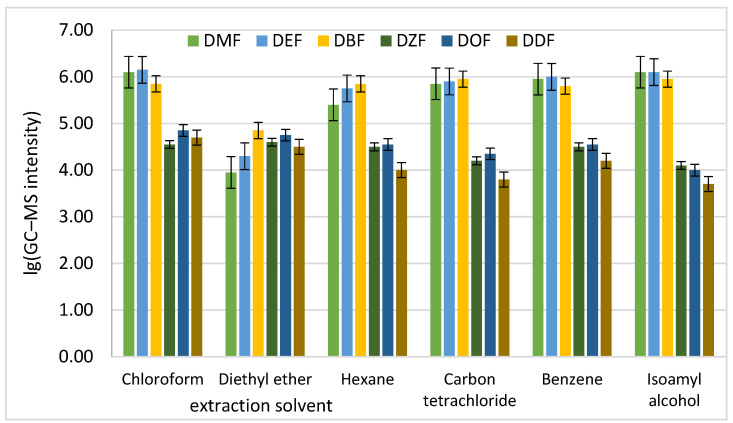
Standard deviations between the concentration values of six phthalates in three parallel extractions.

The precision of the extraction procedure was evaluated through three parallel extractions, as shown in Figure 2.

As shown in Figure 2, the lowest standard deviations were observed for chloroform and carbon tetrachloride, confirming the good reproducibility of these extractants.

Considering both analytical performance and economic aspects, chloroform was selected as the optimal extraction solvent for further method development.

### 3.6. Optimization of Extraction Parameters

**Figure 3:**
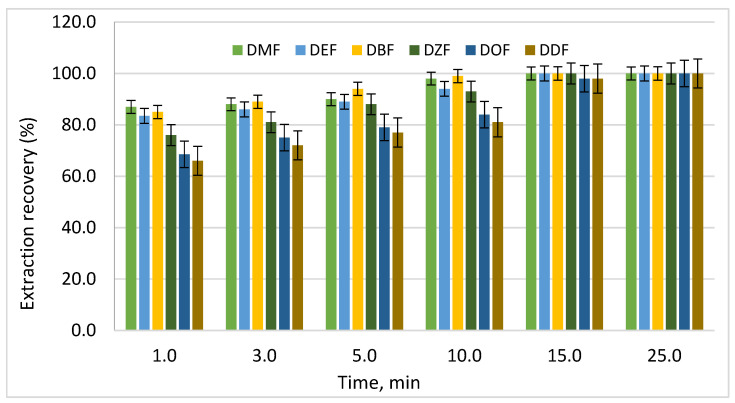
Effect of extraction time on phthalate recovery from the M-15 model solution using chloroform. Recovery at 25 min was taken as 100%.

After selecting the extraction solvent, the extraction procedure was optimized in order to maximize analyte recovery while maintaining a simple and reproducible analytical workflow. To determine the optimal extraction time required to reach equilibrium between the aqueous phase and the organic extractant, kinetic experiments were conducted using the selected chloroform–solution system. The results are presented in the histogram shown in Figure 3.

The extraction process was monitored for up to 25 min, with phthalate recovery at 25 min considered as the reference equilibrium value (100%). The extraction time significantly influenced phthalate recovery. The results showed that between 68% and 87% of the total extractable phthalates were recovered within the first minute of extraction, indicating rapid mass transfer between phases.

Increasing the extraction time resulted in a gradual increase in analyte recovery, indicating that equilibrium between the beverage matrix and the organic solvent had not yet been reached during shorter extraction intervals. However, extending the extraction time beyond the optimal value did not significantly improve recovery, suggesting that equilibrium conditions had already been achieved.

The solvent-to-sample ratio also played an important role in the extraction process. Increasing the solvent volume enhanced mass transfer between phases and improved analyte extraction. Nevertheless, excessively large solvent volumes did not provide significant additional benefits and would negatively affect method sustainability. Therefore, an optimal solvent-to-sample ratio of 1:10 was selected to balance analytical performance and solvent consumption.

These observations are consistent with previous analytical studies on phthalate extraction from food and beverage matrices, where extraction efficiency was primarily controlled by solvent polarity and phase partition equilibrium.

### 3.7. Influence of pH, Alcohol Content, and Sugar Content

The composition of beverage matrices may influence both analyte stability and extraction efficiency. Therefore, the influence of pH, alcohol content, and sugar concentration was systematically investigated.

#### 3.7.1. Effect of pH

The pH of the sample matrix may influence both the stability of phthalate esters and their extraction efficiency. Therefore, the dependence of phthalate recovery on pH was investigated using a series of aqueous-alcoholic solutions (15% *v*/*v* ethanol) with pH values ranging from 3.0 to 7.0.

The tested solutions had the following measured pH values: 2.99, 3.58, 4.14, 4.53, 4.99, 5.44, 6.06, 6.48, and 6.98. They were prepared from an aqueous-alcoholic mixture by adding calculated amounts of 0.1 M tartaric acid solution. The selected pH range corresponds to typical acidity values of beverage matrices as well as to the pH of deionized water obtained by reverse osmosis (approximately 6.5–7.0).

**Figure 4:**
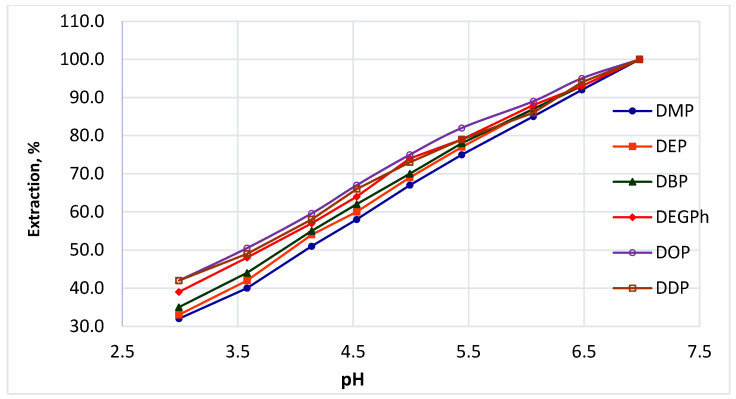
Effect of pH on the extraction recovery of six phthalates from the model solution. Initial concentrations: DMP 0.106 mg/L, DEP 0.102 mg/L, DBP 0.108 mg/L, DEHP 0.105 mg/L, DOP 0.092 mg/L, and DDP 0.096 mg/L.

The initial concentrations of phthalates in the model solutions were: DMP 0.106 mg/L, DEP 0.102 mg/L, DBP 0.108 mg/L, DEHP 0.105 mg/L, DOP 0.092 mg/L, and DDP 0.096 mg/L. Figure 4 indicates that moderate variations in pH did not significantly affect phthalate recovery. The extraction efficiency remained stable across the investigated pH range. No evidence of hydrolysis of phthalate esters was observed under these conditions.

These findings suggest that phthalates are relatively stable in slightly acidic to neutral aqueous-alcoholic systems, and that pH does not represent a critical parameter for their extraction within typical beverage conditions.

**Figure 5:**
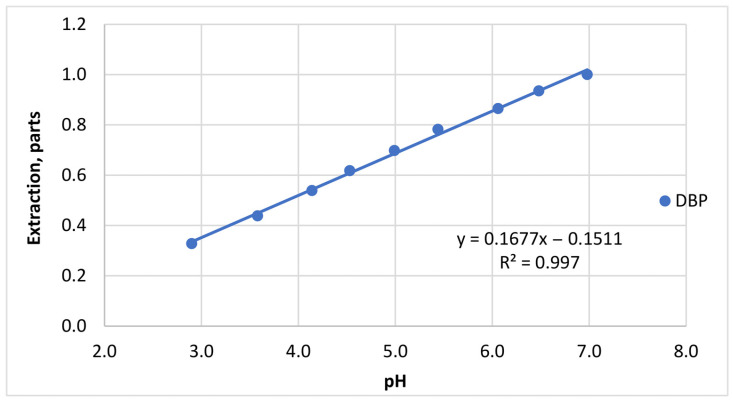
First-order approximation of the effect of pH on phthalate extraction efficiency (DBP as an example).

Correction factors for chemical parameters were calculated using first-order approximations (f(pH), f(C_sugar_), f(C_alcohol_)), with DBP as an example, as in Figure 5.

These results indicate that phthalate esters remain relatively stable in slightly acidic to neutral aqueous-alcoholic matrices, which is consistent with previously reported studies on phthalate migration and stability in beverage systems [71].

The limited influence of pH on extraction efficiency may be explained by the hydrophobic nature of phthalate esters, which reduces their sensitivity to moderate pH variations in aqueous systems.

#### 3.7.2. Effect of Alcohol Content

**Figure 6:**
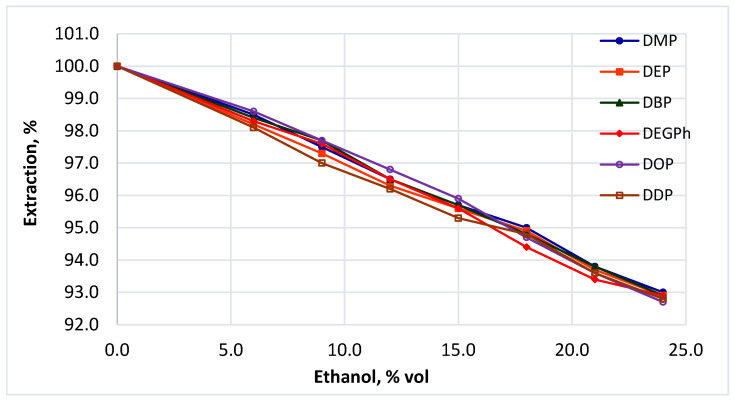
Recovery of six phthalates as a function of ethanol concentration in aqueous-alcoholic solutions.

Alcoholic components in beverages can influence the partitioning of hydrophobic contaminants such as phthalates. Ethanol increases the solubility of these compounds in the aqueous matrix, potentially enhancing their transfer to the extraction solvent. To evaluate this effect, aqueous-alcoholic solutions were prepared with ethanol concentrations of 0, 6, 9, 12, 15, 18, 21, and 24% *v*/*v*, representing water, low-alcohol, and fortified beverages. Initial phthalate concentrations were: DMP 0.106 mg/L, DEP 0.102 mg/L, DBP 0.108 mg/L, DEHP 0.105 mg/L, DOP 0.092 mg/L, and DDP 0.096 mg/L. The results (Figure 6) show that increasing alcohol content slightly improved phthalate recovery. This effect can be attributed to enhanced solubility and mobility of hydrophobic compounds in the aqueous-alcoholic matrix, facilitating their transfer into the organic extraction phase.

However, the observed influence was relatively minor, indicating that alcohol concentration does not represent a critical factor affecting extraction efficiency within the investigated range.

Similar matrix effects have been reported in studies on phthalate migration from plastic packaging into alcoholic beverages [56], supporting the observed trend.

#### 3.7.3. Effect of Sugar Content

Sugar concentration influences the viscosity of beverage matrices and may therefore affect analyte diffusion during extraction. Higher sugar concentrations resulted in slightly lower recoveries, most likely due to reduced mass transfer in more viscous solutions.

**Figure 7:**
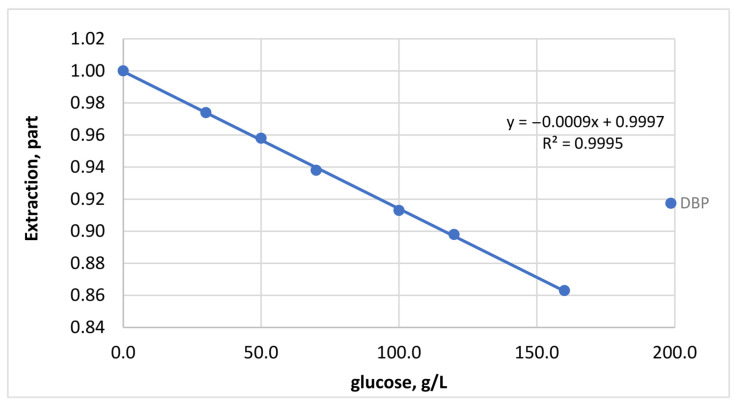
Characteristics of the first-order approximating curve representing the general trend of phthalate extraction from solutions with varying glucose content (using DBP as an example).

The effect of sugars on phthalate recovery was studied using aqueous-alcoholic solutions with a pH of 3.0 (Figure 7). Glucose (crystalline, reagent grade) was used to prepare contaminated solutions. Concentrations of 0.0, 30.0, 50.0, 70.0, 100.0, 120.0, and 160.0 g/dm^3^ were obtained by adding the calculated amount of crystalline glucose during solution preparation.

Despite this matrix effect, the developed extraction method remained sufficiently robust for the analysis of beverages with different sugar contents.

### 3.8. Quantification Procedure

Quantitative determination of phthalates was performed using calibration curves constructed within an appropriate concentration range. The calibration plots showed excellent linearity, with correlation coefficients (R^2^) higher than 0.99, confirming the reliability of the analytical response.

To determine the calibration parameters, the chromatograph was calibrated using the internal standard method. The response factor (k) was calculated according to Equation (1):

(1) $$k=\frac{S_{st}^{is}}{S_{st}^{DBP}}\cdot \frac{n_{st}^{DBP}}{n_{st}^{is}}$$

where *k* is the response factor, $S_{st}^{is}$ is the peak area of the internal standard, $S_{st}^{DBP}$ is the area of the DBP standard, $n_{st}^{DBP}$ is the amount of the injected DBP standard, $n_{st}^{is}$ is the amount of the internal standard.

The amount of analyte in the sample (DBP in this case) was calculated using Equation (2):

(2) $$n_{an}^{DBP}=k\cdot \frac{n^{is}}{S^{is}}\cdot S_{an}^{DBP}$$

where $n_{an}^{DBP}$ is the content of phthalate in the sample being analyzed, $S_{an}^{DBP}$ is the DBP peak area in the sample being analyzed, $S^{is}$ is the DBP peak area of the internal standard added to the sample, and $n^{is}$ is the amount of internal standard added to the sample being analyzed.

**Figure 8:**
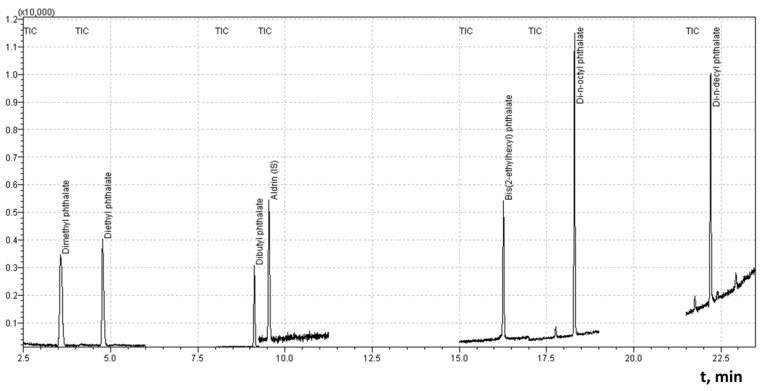
Chromatogram of phthalate solution (DMP 0.103 mg/L, DEP 0.110 mg/L, DBP 0.098 mg/L, DEHP 0.116 mg/L, DOP 0.121 mg/L and DDP 0.118 mg/L) with aldrin as IS (0.50 mg/L).

In the practical part of the analytical work, aldrin was used as an internal standard (Figure 8).

The selection of aldrin as an internal standard was based on several considerations: (i) its availability in many analytical laboratories performing environmental or food monitoring; (ii) the expected absence of aldrin residues in environmental samples due to restrictions imposed by the Stockholm Convention on Persistent Organic Pollutants; (iii) previously reported absence of aldrin residues in soils of the Republic of Moldova [72,73], and (iv) a retention time similar to that of DBP, Figure 7.

In contrast, the use of benzyl benzoate as an internal standard, as reported in several studies [74,75], may be less reliable due to its widespread application in pharmaceutical and cosmetic products [76], which increases the risk of background contamination.

Phthalate concentrations were calculated after computer integration of chromatographic peaks corresponding to the selected *m*/*z* values. The average analyte concentration and the relative difference between two parallel measurements were calculated according to Equation (3):

(3) $$C=\frac{1}{i}\cdot \sum_{i}C_{i},\;C_{1}-C_{2}\leq 0.25\cdot \bar{C}$$

The analytical characteristics obtained during method validation are summarized in Table 3.

Limits of detection (LOD) and limits of quantification (LOQ) were estimated using the signal-to-noise ratio approach. The obtained values demonstrate that the proposed analytical procedure provides sufficient sensitivity for the determination of trace levels of phthalates in beverage matrices.

Overall, the analytical performance of the developed method is comparable with previously reported chromatographic procedures used for the determination of phthalates in food and beverage samples.

### 3.9. Method Validation and Interlaboratory Study

The developed analytical method was validated in terms of linearity, recovery, precision, and sensitivity. Calibration curves demonstrated excellent linearity within the investigated concentration ranges, with correlation coefficients (R^2^) higher than 0.99 for all analytes.

The recovery of DBP, used as a representative compound of the phthalate homologous series, ranged from 81 to 90% for low-alcohol beverages and wines (0–24% *vol.* ethanol) and from 88 to 96% for strong alcoholic beverages (25–95% *vol.* ethanol). These recovery values indicate satisfactory extraction efficiency for phthalates across different beverage matrices.

Method precision was evaluated in terms of repeatability and reproducibility. The relative standard deviation under repeatability conditions (RSDr) did not exceed 5%, while interlaboratory repeatability (RSDL) ranged between 7 and 13%. The reproducibility (RSDR) values were approximately 15%, confirming the robustness of the proposed analytical procedure.

The LOD ranged from 0.006 to 0.01 mg/L, whereas the LOQ was between 0.02 and 0.03 mg/L. These values demonstrate that the developed method provides sufficient sensitivity for the determination of trace concentrations of phthalates in beverage samples.

Overall, the analytical performance obtained in this study is comparable with previously reported chromatographic methods used for the determination of phthalates in food and beverage matrices, confirming the suitability of the proposed procedure for routine analytical applications.

The interlaboratory tests were carried out within the framework of an international study involving 15 accredited laboratories, aimed at developing a GC–MS procedure for the determination of phthalate residues using deuterated internal standards (DBP-d_4_ and DEHP-d_4_).

The results were evaluated using z-score statistics, which are commonly applied in interlaboratory proficiency testing. The obtained values showed that most laboratories achieved Z| ≤ 2, corresponding to satisfactory analytical performance and confirming the reliability and reproducibility of the proposed method across different laboratories.

The proposed analytical approach combines a simple extraction procedure with satisfactory analytical sensitivity and reproducibility, making it suitable for routine monitoring of phthalate contamination in alcoholic beverages.

### 3.10. Development of a Procedure for a Comprehensive Assessment of Phthalate Content

#### 3.10.1. Hydrolysis-Based Approach

Differences in maximum permissible concentrations (MPCs) for phthalates across various regulatory systems, together with the susceptibility of phthalate esters to physicochemical and biological degradation, complicate the reliable assessment of phthalate contamination in beverages and environmental samples [77].

To address these inconsistencies, a unified analytical approach for evaluating total phthalate content in aqueous-alcoholic matrices was developed. The proposed procedure is based on the hydrolysis of phthalate esters in the test sample, followed by the quantitative determination of *o*-phthalic acid, which represents a common structural fragment of all phthalate esters.

During hydrolysis, phthalate esters are converted into the corresponding alcohols and *o*-phthalic acid. Therefore, the concentration of *o*-phthalic acid formed after saponification can be used as an indicator of the total content of phthalate esters and their transformation products in the analyzed sample.

**Figure 9:**
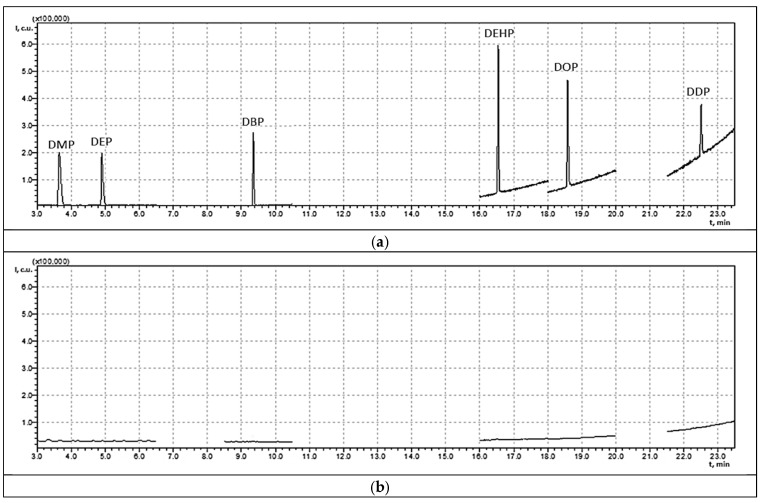
GC–MS chromatograms of a contaminated aqueous-alcoholic solution containing DMP (1.082 mg/L), DEP (1.075 mg/L), DBP (1.112 mg/L), DEHP (1.157 mg/L), DOP (1.133 mg/L), and DDP (1.140 mg/L): (**a**) original solution; (**b**) neutralized solution after hydrolysis.

The completeness of phthalate hydrolysis was verified using GC–MS analysis by comparing chromatograms of the original solution and the hydrolyzed sample (Figure 9).

The disappearance of the characteristic phthalate peaks after hydrolysis confirmed the completeness of ester saponification and the formation of the corresponding hydrolysis products.

#### 3.10.2. Determination of *O*-Phthalic Acid

Following hydrolysis, the concentration of *o*-phthalic acid was determined using capillary electrophoresis with spectrophotometric detection. This technique provides high separation efficiency and sensitivity for the determination of small organic acids in aqueous matrices.

The limit of quantification (LOQ) for *o*-phthalic acid was calculated based on the signal-to-noise ratio and was found to be 0.067 mg/L, where N represents the noise level of the baseline signal measured in a blank sample.

Capillary electrophoresis and high-performance liquid chromatography (HPLC) have been reported as suitable analytical techniques for the determination of *o*-phthalic acid, whereas gas chromatography generally requires derivatization or the use of specialized capillary columns [45].

#### 3.10.3. Validation of the Capillary Electrophoresis Method

The analytical characteristics of the proposed capillary electrophoresis procedure were evaluated to assess its applicability for the determination of *o*-phthalic acid formed during phthalate hydrolysis. The main validation parameters are summarized in Table 4.

#### 3.10.4. Analytical Application

The results obtained in this study demonstrate that the total phthalate content in beverages can be estimated as the sum of phthalate esters expressed in terms of *o*-phthalic acid concentration.

The experimental work also allowed the determination of the amount of alkali required for the complete saponification of phthalate esters in aqueous-alcoholic media. Based on these results, the analytical parameters and validation criteria for the proposed hydrolysis–capillary electrophoresis approach were established.

Overall, the developed method provides a complementary tool for the comprehensive assessment of phthalate contamination in beverage matrices.

## 4. Conclusions

In this study, an analytical procedure for the determination of phthalates in aqueous-alcoholic matrices was developed and validated. The proposed method combines solvent extraction, gas chromatography–mass spectrometry (GC–MS) analysis, and a complementary hydrolysis approach for the comprehensive assessment of total phthalate content.

The optimization of extraction parameters, including solvent type, extraction time, pH, alcohol content, and sugar concentration, demonstrated that these factors have only a limited influence on phthalate recovery within the investigated ranges. The developed extraction procedure showed satisfactory analytical performance, with recoveries ranging from 81–96%, repeatability values below 5%, and limits of detection between 0.006 and 0.01 mg/L.

To address the challenges associated with the determination of multiple phthalate esters and their degradation products, a complementary analytical strategy based on hydrolysis and determination of *o*-phthalic acid was proposed. This approach enables the estimation of the total phthalate content in samples by converting individual esters into a single measurable compound. The determination of *o*-phthalic acid using capillary electrophoresis demonstrated suitable analytical performance, with a linearity range of 0.1–5.0 mg/L and a limit of quantification of 0.07 mg/L.

The results obtained confirm that the proposed analytical methodology provides a reliable determination of phthalate contamination in beverage matrices. The combined chromatographic and hydrolysis–electrophoretic approaches may serve as a useful tool for routine monitoring and comprehensive assessment of phthalate residues in aqueous-alcoholic systems and non-alcoholic beverages.

Future Work. The proposed methodology was successfully validated and implemented as a specific procedure in a food quality and safety testing laboratory. Further evaluation will be carried out using a hydrolysis-based approach to determine total phthalate content relative to individual PAE concentrations, enabling assessment of the method’s robustness.

## Figures and Tables

**Table 1:** Migration of potentially toxic compounds from plastic (PET) food packaging intended for water, soft drinks, and edible vegetable oils.

Water, Non-Alcoholic Beverages *							
**Code**	Product Name	V, L	PET Color	HDPE Color (Cap)	Quantified NIAS, mg/L		NIAS, Traces
					DBP	DEHP	
A1	OM (carbonated water)	0.50	light blue	light blue	0.054 ± 0.007	≥0.005	5-hexene-1,3,5-triyltribenzene (styrene trimer)
A2	OM (still water)	0.50	light blue	light blue	0.006 ± 0.002	≥0.005	5-hexene-1,3,5-triyltribenzene (styrene trimer)
A3	Cappy Pulpy	0.33	gray	orange	0.048 ± 0.008	0.026 ± 0.003	5-hexene-1,3,5-triyltribenzene (styrene trimer)
							Erucylamide
A4	Bucovina Fructata	0.50	green	green	0.051 ± 0.005	≥0.005	-
A5	Davidan (still water)	0.50	blue	blue	0.006 ± 0.005	0.007 ± 0.003	-
A6	Fuzetea	0.50	colorless	green	0.042 ± 0.006	0.028 ± 0.003	5-hexene-1,3,5-triyltribenzene (styrene trimer)
							Erucylamide
A7	Dorna (still water)	0.50	blue-gray	green	≥0.005	≥0.005	5-hexene-1,3,5-triyltribenzene (styrene trimer)
							Erucylamide
Edible vegetable oils **							
U1	Bunetto (sunflower oil)	0.95	colorless	yellow	0.048 ± 0.007	0.034 ± 0.016	Phosphoric acid, tris (2-ethylhexyl) ester
U2	Sloboda (sunflower oil)	1.00	colorless	red	0.076 ± 0.008	0.009 ± 0.006	-
U3	Oleina (sunflower oil)	0.85	colorless	yellow	0.086 ± 0.009	0.012 ± 0.007	-
U4	Floris (sunflower oil)	0.95	colorless	red	0.092 ± 0.012	0.082 ± 0.006	Butyl 2-ethylhexyl phthalate
							Bis(2-ethylhexyl) adipate
							Mono(2-ethylhexyl) phthalate
							1,1,3-Trimethyl-3-phenylindane 80%
U5	Beauty Aldero (Corn oil)	0.80	colorless	green	0.064 ± 0.011	0.038 ± 0.004	-
U6	Sloboda (sunflower oil)	1.00	colorless	green	0.072 ± 0.009	0.011 ± 0.007	-

* Migration duration: 30 days at room temperature. ** Migration duration—14 days at room temperature.

**Table 2:** Signal intensities (chromatograph mass spectrometric peak areas) by *m*/*z* sum for phthalates in the corresponding extractants.

Extractant	DMP, 0.106 mg/L	DEP, 0.102 mg/L	DBP, 0.108 mg/L	DEHP, 0.105 mg/L	DOP, 0.092 mg/L	DDP, 0.096 mg/L
Chloroform	1,457,312	1,400,556	749,389	31,858	66,574	46,289
Diethyl ether	8644	18,889	73,472	33,630	53,973	28,596
Hexane	225,440	626,408	702,451	28,768	32,781	10,158
Carbon tetrachloride	729,997	798,685	818,517	16,099	21,038	5506
Benzene	868,555	980,014	592,497	30,551	31,141	14,750
Isoamyl alcohol	1,253,308	1,232,489	899,266	11,150	9768	5084

**Table 3:** Analytical characteristics of the DBP analysis procedure in low-alcohol beverages, wines and strong drinks obtained during method validation.

Analytical Characteristic	Low-Alcohol Beverages, Wines (0–24% vol. Ethanol)	STRONG Drinks (25–95% vol. Ethanol)
Linear range (mg/L)	0–0.50	0–1.00
Recovery (%)	C = 0.1 mg/L—81% C = 0.2 mg/L—88% C = 0.3 mg/L—90%	C = 0.1 mg/L—96% C = 0.2 mg/L—88% C = 0.3 mg/L—92%
Repeatability (RSDr, %)	5	5%
Intermediate precision (RSDi, %)	13%	7%
Reproducibility (RSDR, %)	15%	15%
Repeatability limit (r)	0.10X	0.10X
Reproducibility limit (R)	0.25X	0.25X
Limit of detection (LOD, mg/L)	0.006	0.01
Limit of quantification (LOQ, mg/L)	0.02	0.03

RSDr—relative standard deviation under repeatability conditions; RSDi—relative standard deviation for intermediate precision; RSDR—relative standard deviation under reproducibility conditions.

**Table 4:** Analytical performance characteristics of the capillary electrophoresis method for the determination of *o*-phthalic acid.

Analytical Characteristic	Value
Linear range (mg/L)	0.1–5.0 mg/L
Recovery (%)	68–82%
Repeatability (RSDr, %)	8%
Intermediate precision (RSDi, %)	15%
Reproducibility (RSDR, %)	18%
Repeatability limit (r)	0.25X
Reproducibility limit (R)	0.4X
Limit of detection (LOD, mg/L)	0.02 mg/L
Limit of quantification (LOQ, mg/L)	0.07 mg/L

## Data Availability

The original contributions presented in this study are included in the article. Further inquiries can be directed to the corresponding author.
